# Protocol of the process evaluation of cluster randomised control trial for estimating the effectiveness and cost-effectiveness of a complex intervention to increase care home staff influenza vaccination rates compared to usual practice (FluCare)

**DOI:** 10.1186/s13063-023-07613-5

**Published:** 2023-09-15

**Authors:** Linda Birt, Thando Katangwe-Chigamba, Sion Scott, David J Wright, Adam P. Wagner, Erika Sims, Veronica Bion, Carys Seeley, Faisal Alsaif, Allan Clarke, Alys Griffiths, Liz Jones, Alison Bryant, Amrish Patel

**Affiliations:** 1 School of Healthcare, University of Leicester, Leicester, UK; 2 Norwich Clinical Trials Unit, University of East Anglia, Norwich, UK; 3 National Institute for Health Research (NIHR) Applied Research Collaboration (ARC) East of England (EoE), Cambridge, UK; 4 School of Pharmacy, University of East Anglia, Norwich, UK; 5 Norwich Medical School, University of East Anglia, Norwich, UK; 6 Institute of Population Health, University of Liverpool, Liverpool, UK; 7 School of Economics, University of East Anglia, Norwich, UK

**Keywords:** Residential homes, Nursing homes, Care homes, Long-term care facilities, Influenza vaccination, Staff, Employees

## Abstract

**Background:**

Influenza (flu) vaccination rates in UK care home staff are extremely low. Less than 40% of staff in care homes are vaccinated for influenza (flu), presenting risks to the health of frail residents and potential staff absence from cross-infection. Staff often do not perceive a need for vaccination and are unaware they are entitled to free flu vaccination. The FluCare study, a cluster randomised control trial (RCT), uses behavioural interventions to address barriers. Videos, posters, and leaflets are intended to raise awareness of flu vaccination benefits and debunk myths. On-site staff vaccination clinics increase accessibility. Financial incentives to care homes for improved vaccination rates and regular monitoring influence the environment. This paper outlines the planned process evaluation which will describe the intervention’s mechanisms of action, explain any changes in outcomes, identify local adaptations, and inform design of the implementation phase.

**Methods/design:**

A mixed method process evaluation to inform the interpretation of trial findings.

**Objectives:**

• Describe the intervention as delivered in terms of dose and fidelity, including adaptations and variations across care homes.

• Explore the effects of individual intervention components on primary outcomes.

• Investigate the mechanisms of impact.

• Describe the perceived effectiveness of relevant intervention components (including videos, leaflets, posters, and flu clinics) from participant perspectives (care home manager, care home staff, flu clinic providers).

• Describe the characteristics of care homes and participants to assess reach.

A purposive sample of twenty care homes (ten in the intervention arm, ten in the control arm) for inclusion in the process evaluation. Data will include (1) study records including care home site profiles, (2) responses to a mechanism of action questionnaire, and (3) semi-structured interviews with care home staff and clinic providers. Quantitative data will be descriptively reported. Interview data will be thematically analysed and then categories mapped to the Theoretical Domains Framework.

**Discussion:**

Adopting this systematic and comprehensive process evaluation approach will help ensure data is captured on all aspects of the trial, enabling a full understanding of the intervention implementation and RCT findings.

**Trial registration:**

ISRCTN ISRCTN22729870. Registered on 24 August 2022.

**Supplementary Information:**

The online version contains supplementary material available at 10.1186/s13063-023-07613-5.

## Introduction

In the United Kingdom (UK) each year, seasonal influenza (flu) causes around 17,000 deaths [1]. This creates a major risk for older residents of care and nursing homes [2]. Risks can be mitigated by vaccinating care staff [3]. The World Health Organization (WHO) recommends that at least 75% of health and social care staff are vaccinated for flu [4]. In England, rates fall far below this recommendation increasing risks to care home residents’ health and staff well-being. In February 2022, only 26.8% of the total staff of older adult care homes were reported as having received the flu vaccination [5]. Low flu vaccination uptake may in part be due to vaccination hesitancy linked with compulsory COVID-19 vaccinations for care home staff in the UK [6, 7]. However, flu vaccine hesitancy is long-standing, and pre-COVID preparation work for the FluCare study, including a narrative synthesis, survey, and qualitative work, identified five individual-level behavioural barriers to flu vaccination: access to vaccination, cost of vaccination, perceived lack of need, vaccine beliefs, and peer influences. Staff are more likely to take up flu vaccination if they consider it benefits them [8].

**Fig. 1:**
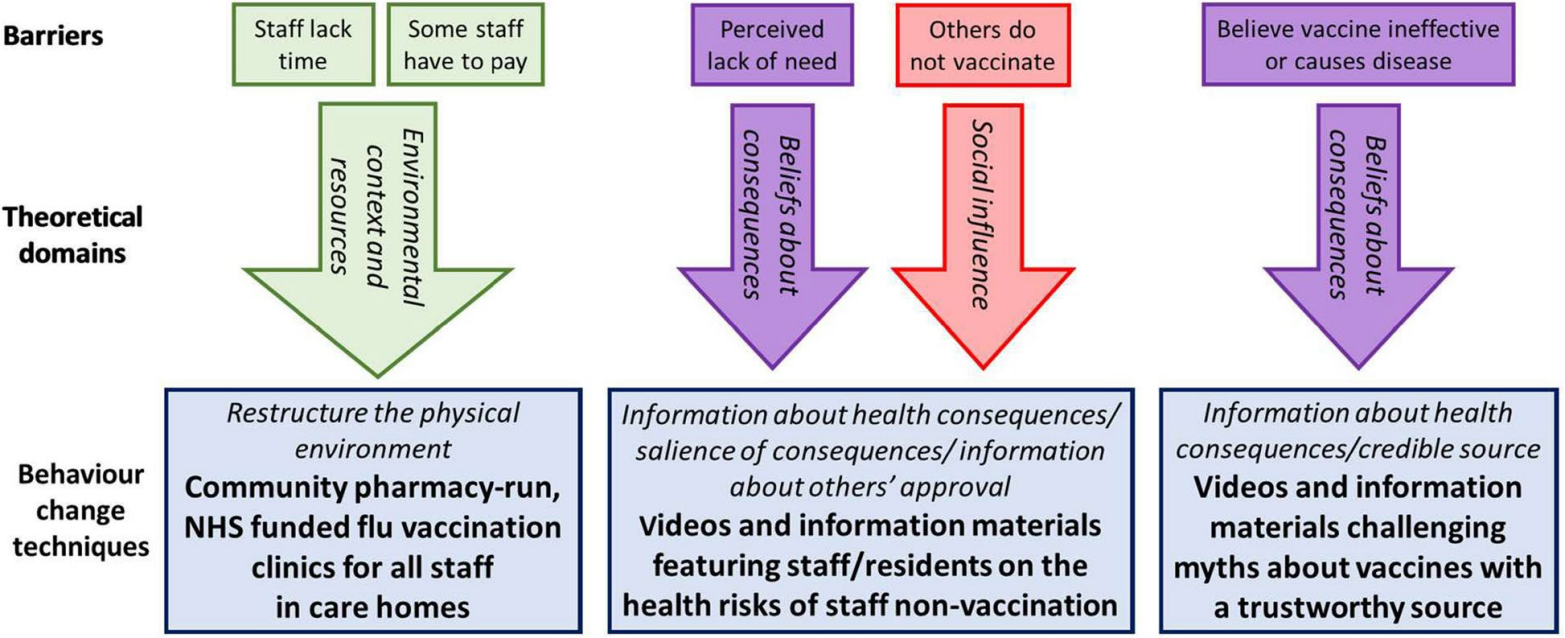
Relationship between behaviour change techniques, barriers, and theory

The FluCare intervention aims to address these barriers by drawing on behavioural change theory. Behaviour change techniques (BCTs) are intended to alter behaviours and are frequently utilised to increase vaccination rates [9]. Mapping known barriers to vaccination to the Theoretical Domains Framework (TDF) informs those domains which require addressing. The TDF is a synthesis of behaviour change theories organised into 14 domains which are the determinants of an individual’s behaviour, including social influence, social/professional role and identity, beliefs about consequences, environmental context, and resources [10]. Using the mapping table by Cane et al. [11], 31 potentially appropriate BCTs, the active ingredients of behaviour change interventions, were identified, see Fig. 1. A nominal group technique stakeholder consensus study [12] with 13 care home staff and managers supported the development of the FluCare intervention elements. Stakeholders selected from the list of BCTs, those which met the APEASE criteria (affordable, practicality, effectiveness, acceptability, side effects, equity) for addressing the barriers [13].

Stakeholders then characterised ways in which each BCT may be operationalised in care home practice. This characterisation was refined by public and patient involvement (care home residents and relatives) to arrive at the following intervention elements.

1. *Restructure the physical environment*—offer NHS-funded flu vaccination clinics in the care home at convenient times to account for shift work. Offer to all staff, including agency, working in the care homes. Flu vaccination clinics run by community pharmacy or GP surgery.
2. *Information about health consequences*—provide information on the health risks of low staff vaccine uptake featuring staff and residents. Information delivered through a short 5-min video and poster. To maximise engagement, the material should reflect staff cultural diversity (i.e. multi-lingual and to represent the range of socio-demographics), particularly given the low vaccine uptake in minority ethnic communities [14].
3. *Information about health consequences from a credible source*—provide information from a trustworthy source, e.g. general practitioner, challenging myths about vaccines such as it causes the flu or being dangerous to pregnant people.

While the FluCare intervention targets staff-level behaviour change, it is widely recognised that a maximising benefit is seen when staff feel the behaviour aligns with the priorities of their organisation. Employer encouragement is a known enabler for staff vaccination [8, 15].

Our intervention (Fig. 1) is therefore augmented by two organisational-level strategies: *regular vaccine uptake monitoring* of care homes and feedback on their uptake performance relative to other care homes.

1. *Financial incentives* for care homes with staff vaccination rate > 70%.

The feasibility study for the FluCare intervention was undertaken during the 2021/2022 flu season, confirming that care homes and vaccination providers (GPs and Pharmacists) could be successfully recruited and were willing to participate [paper in press]. The feasibility study informed the frequency of data collection and design of the control arm. While data collection frequency (monthly versus end of study) did not influence the uptake of flu vaccination in the control arm, monthly data collection was preferred by sites. The provision of posters and leaflets appeared to have a small but limited effect.

A cluster RCT started in October 2022, and recruitment closed on 31 March 2023. The primary outcome measure is the total number of staff vaccinated in a flu season over a total number of staff employed at any point throughout that flu season [16]. Secondary outcome measures are staff flu vaccination rate at the end of November 2022; numbers of staff sick days, GP and nurse visits, and resident hospitalisations; and resident mortality [16].

The definitive RCT and embedded process evaluation was approved by the University of East Anglia ethics committee. The RCT is on the ISRCTN registry (ISRCTN22729870). The process evaluation is embedded in the main RCT protocol version 1.1, 5 August 2022.

## Process evaluation design

A mixed-methods, theory-driven process evaluation will be undertaken in parallel to the FluCare definitive RCT. The evaluation design follows guidance on process evaluations [17]. The Theoretical Domains Framework (TDF) underpins the exploration of the barriers and enablers to flu vaccination uptake within this trial [10]. Consideration will be given to how the intervention might need to be altered to complement current care home systems [18]. The protocol follows SPIRIT guidelines see additional file 1 [19].

## Public involvement

Patient and public involvement members have been involved in all project stages, from inception to providing advice on the different elements of the intervention, e.g. poster and video, providing guidance on how to enhance our approach to recruitment and how to communicate effectively with care homes. They have explored feasibility data and refined interview questions for the process evaluation. They will continue to have regular input into the trial and process evaluation analysis and dissemination.

## Objectives

1. To describe the intervention as delivered in terms of dose and fidelity, including adaptations and variations across care homes
2. To explore the effects of individual intervention components on the primary outcomes
3. To investigate the mechanisms of impact
4. To describe the perceived effectiveness of relevant intervention components (including videos, leaflets, posters, and flu clinics) from participant’s (care home manager, care home staff, and flu clinic providers) perspectives
5. To describe the characteristics of care homes and participants to assess reach

## Process evaluation framework

### Behavioural change intervention

The behavioural assumption is that access to information on the benefits of flu vaccination alongside easy access to vaccination clinics on site will improve staff vaccination rates. Intervention care home managers receive behavioural change information (posters, leaflets, videos). They are asked to make these materials available to all staff, i.e. display posters and leave leaflets in staff rooms etc. and send the video link through the usual means they communicate with staff such as WhatsApp. Care homes will be partnered with a flu vaccination provider: either a pharmacy or GP practice. Flu clinic providers will work with care home managers to deliver up to four vaccination clinics.

The process evaluation will be undertaken at the end of the trial. Data will be collected and analysed to understand and provide an explanation for trial outcomes and inform future adaptations considering the four aspects of implementation, mechanisms of action, outcomes, and contextual factors.

### Trail status

Recruitment to the process evaluation commenced when the RCT ended on 1 May 2023 and recruitment will close on 30 July 2023. The process evaluation is embedded in the main RCT protocol version 1.1, 5 August 2022.

### Sample

Seventy-five care homes were randomised in the RCT: 38 to the control arm and 37 to the intervention arm. The process evaluation data will be from a sample of 10 control and 10 intervention care homes purposefully selected for variety in characteristics, namely the size of the home, characteristics of the staff, and type of care home registration. Up to 20 flu clinic providers will be invited, including those unable to deliver a flu clinic. Recruitment for the process evaluation will close on 30 July 2023.

### Data collection and analysis

Data will be collected and analysed to provide evidence for each aspect of the process evaluation. *Implementation*: information on the use of intervention material to assess reach, dose, and fidelity of the intervention, see Table 1.

**Table 1:** Intervention implementation tasks and data collection during the process evaluation

Task	Aim (what is being assessed)	Data collected	Date source
Provision of behavioural change material leaflets (posters) to all care home staff	Reach intervention	Where leaflets and posters displayed Staff awareness of seeing posters, leaflets	Care home manager interviews Staff interviews
Provision of behavioural change video to all staff	Reach and dose of intervention	Number of times the video played Staff awareness of video	Metrics on viewing (which is embedded in video) Staff interviews
Provision of on-site flu vaccinator clinic	Dose and fidelity to intervention	Number of flu clinics provided	Flu vaccination clinic log Interviews

*Mechanisms of impact*: Data will be collected to understand the mechanisms of impact in achieving the aims of changing staff beliefs about flu vaccination and increasing staff vaccination rates. Data on vaccination rates in both arms will help identify if the intervention increases flu vaccination rates over usual patterns of change due to factors outside the trial such as public health campaigns, see Table 2.

**Table 2:** Mechanism of impact and data collection in process evaluation

Impact	Mechanism of impact	Data collected	Date source
Improved staff understanding of the benefits of flu vaccination for themselves	Provide behavioural change material (videos, leaflets, posters) to all care home staff	Staff perceptions of material Change in staff beliefs and intention to be vaccinated	Semi-structured interviews with all care home staff Pre- and post-intervention mechanisms of action survey
Improved access to flu vaccinations	Provide onsite flu vaccination clinics	Number of staff who attended clinic and had vaccination Number of staff who attended clinic and refused vaccination Staff perceptions of the flu clinics	Flu vaccination clinic log Flu vaccination clinic log Staff interviews included those who did and did not receive vaccination at the flu clinic

*Contextual factors* as characteristics of care homes and their staff and availability of flu vaccination clinics will be considered on how they affect delivery and intervention’s impact of the intervention, see Table 3.

**Table 3:** Contextual factors and data collected as part of process evaluation

Contextual factors	To examine	Data collected	Data source
Barriers to delivering the intervention	Staff variation	Staff perceptions	Care home staff interviews Flu clinic provider interviews
Facilitators to delivering the intervention		Staff perceptions	Care home staff interviews Flu clinic provider interviews
Site factors	Inter-site variation	Care home factors	Baseline and end of study Site profile questionnaire
		Care home staff factors	Care home staff logs
		Flu clinic provider characteristics	Flu clinic provider logs
Statutory public health policies	Variation in national vaccination communication		Review of public-facing documents

### Data source

#### Site profile questionnaire and care home logs

These are records generated during the trial. All homes will be characterised at the start and end of the trial period to identify the characteristics (i.e. home type (private/charity/local authority), size (beds), with/without nursing, number and type of staff (age gender and ethnicity), staff employment status (employed, bank, agency voluntary), infection control policies, protocols/operating procedures, vaccination policy, guidance/education routinely provided) and changes which may affect the intervention implementation during the trial period. Descriptive narrative analysis of data will provide context to any variation in implementation and outcomes.

#### Mechanism of Action Questionnaire (MAQ)

The MAQ comprises of four items, each with a 5-point Likert scale response option (strongly disagree to strongly agree), measuring the extent to which the intervention has addressed the four theoretical domains (Fig. 1). Managers in a sample of 20 care homes (control *n* = 10, intervention *n* = 10) will be asked to distribute the MAQ electronically to all staff at baseline; those responding will be invited to complete the MAQ again at the end of the intervention period. Data will be informally compared using descriptive statistics for each respondent to the extent to which the intervention has addressed the barriers to flu vaccination. Variations in MAQ responses between participants, care homes, and other contextual factors will be explored further using qualitative interviews.

#### Semi-structured interviews

At the end of the intervention period, care home managers, their staff, and vaccination providers will be invited to an online interview. We will seek to interview up to 13 care home managers (control *n* = 3, intervention *n* = 10). Interviews will focus on the implementation and perceived outcomes of the intervention and clarify contextual information. We will seek to interview up to 30 care home staff (control *n* = 4, intervention *n* = 26) sampled for a variety of job roles. Interviews will focus on thoughts about flu vaccination, access to intervention material, use, or non-use of flu vaccination clinics. We will see to interview up to 20 vaccination providers including those who did not deliver flu vaccination clinics. Interviews will focus on the implementation of the flu clinic, outcomes of running the clinic, and contextual factors. We are not interviewing residents or their families as they are not directly involved in the study.

Each participant will be provided with a participant information sheet and electronic written consent taken prior to the interview. Interview topic guides have been designed with patient and public involvement representatives and seek to ask staff for views on how each BCT was delivered (content); its acceptability, including how compatible it was with routine practices and how each BCT worked within the home (theoretical fidelity), and exploring why BCTs have succeeded in/failed to address certain barriers. Interviews with care home managers will focus on procedures for vaccination clinic visits, staff working arrangements, local infection control policies, and other contextual issues affecting intervention delivery. Interviews with pharmacists/healthcare practitioners delivering flu vaccination clinics will elicit experiences of setting up/running flu vaccination visits and interviews with pharmacists/healthcare practitioners who were unable to deliver on-site clinics will explore the barriers and challenges that prevented them from providing the service. All interviews will be audio-recorded and transcribed verbatim and last no longer than 60 min. At this point, all identifiers will be removed and transcriptions checked against the audio recording. Anonymised transcripts will be uploaded to NViVO for analysis. Audio recordings will be destroyed after analysis. Following an inductive thematic analysis of interview transcripts, each of them will be mapped across to the TDF, to examine how the process and content of the intervention functioned from the participants’ perspective, identifying how barriers were overcome to increase vaccination rates and intervention sustainability over time.

#### Documentary review of policies and protocols

Relevant protocols, policies, and standard operating procedures (e.g. national flu campaign policies; infection control procedures) will be reviewed to understand which guidance for flu vaccinations is in place at the time of the intervention and how they are operationalised within each home, providing context to the analyses.

### Data synthesis

Once all process and main trial outcomes are reported, all data sets will be integrated using a triangulation approach to consider agreement, partial agreement, silence, and dissonance across the data [20]. This will identify, clarify, and relate causal pathways related to participant experience, providing a means to explain unexpected outcomes and identify optimal intervention contexts. If the FluCare trial findings suggest that behaviour change strategies are effective in increasing care home staff vaccinations, the process evaluation will inform recommendations for implementation into routine practice. The process evaluation is designed to enable an understanding of the reasons for where the intervention has, or has not, been successful. The FluCare study has a discrete programme of work to consider how to disseminate learning and co-design resources for use in practice, which will take place following completion of the RCT.

## Discussion

This study is one of the first to use a theory-informed intervention designed to comprehensively address identified barriers to care home staff influenza vaccination. This intervention has five distinct elements designed to increase staff awareness of the benefits of vaccination, make vaccination easier to access, and develop a workplace culture that prioritises flu vaccination. The five elements include written information through posters and leaflets, visual information in video, flu vaccination clinics on site, regular monitoring and feedback on flu vaccination rates, and financial incentives for achieving vaccination rates of 70%. Our feasibility work indicated that not all intervention elements were delivered; for example, the videos were not viewed. Purposive sampling will provide cohorts for comparison, examining if organisational culture in support of vaccination is sufficient to increase vaccination rates in care home staff.

In summary, results from the process evaluation will provide evidence of the efficacy of the behavioural change strategies within the complexity of care homes providing care to older people across England. Understanding how this theoretically informed intervention works within a real-world setting is important to enable recommendations for upscaling of behavioural change strategies. If the RCT does not find statistically significant differences between the intervention and control arms, the process evaluation will provide explanations for this and importantly expose which distinct elements work and in what ways. The final work programme in FluCare will draw on the principles of the RE-AIM framework [21] to develop recommendations for wider implementation.

### Supplementary Information

**Additional file 1.**

## Data Availability

Requests for access to the FluCare trial dataset should be made to Dr. Amrish Patel (Amrish.Patel@uea.ac.uk) and/or Researchsponsor@uea.ac.uk. During the trial requests will be reviewed by the chief investigators, trial management group, and the trial steering committee. Post-trial requests will be reviewed by the chief investigators (and representatives from the trial management group where available) and sponsor. Requests will be reviewed to ensure that there is no conflict with the objectives of the FluCare trial (pre-publication of FluCare findings) or funder constraints. All data requests will be subject to a data sharing agreement with the University of East Anglia.

## Abbreviations

BCT: Behavioural change strategy
GP: General practitioner
MAQ: Mechanism of Action Questionnaire
SPQ: Site Profile Questionnaire
TDF: Theoretical Domains Framework
WHO: World Health Organization
UK: United Kingdom

## References

[1] Wendelboe AM, Grafe C, McCumber M, Anderson MP (2015). Inducing herd immunity against seasonal influenza in long-term care facilities through employee vaccination coverage: a transmission dynamics model. Comput Math Methods Med.

[2] Van Den Dool C, Bonten MJM, Hak E, Heijne JCM, Wallinga J (2008). The effects of influenza vaccination of health care workers in nursing homes: insights from a mathematical model. PLoS Med.

[3] Ng ANM, Lai CKY (2011). Effectiveness of seasonal influenza vaccination in healthcare workers: a systematic review. J of Hosp Infect.

[4] World Health Assembly Resolution WHA56.19. Prevention and control of influenza pandemics and annual epidemics. In: Fifty-sixth World Health Assembly, Geneva, 28 May, 2003. Geneva: World Health Organization; 2003. http://apps.who.int/gb/archive/pdf_files/WHA56/ea56r19.pdf

[5] Department Health and Social Care. Adult Social Care Monthly Statistics England: March 2022. https://www.gov.uk/government/statistics/adult-social-care-in-england-monthly-statistics-march-2022/adult-social-care-monthly-statistics-england-march-2022

[6] Kumar S, Shah Z, Garfield S (2022). Causes of vaccine hesitancy in adults for the influenza and COVID-19 vaccines: a systematic literature review. Vaccines.

[7] Troiano G, Nardi A (2021). Vaccine hesitancy in the era of COVID-19. Public Health.

[8] Shroufi A, Copping J, Musonda P, Vivancos R, Langden V, Armstrong S, Slack R (2009). Influenza vaccine uptake among staff in care homes in Nottinghamshire: a random cluster sample survey. Public Health.

[9] Attwell K, Betsch C, Dubé E, Sivelä J, GagneurA, Suggs LS, Picot V, Thomson A. Increasing vaccine acceptance using evidence-based approaches and policies: insights from research on behavioural and social determinants presented at the 7th Annual Vaccine Acceptance Meeting. Int J Infect Dis. 2021;105:188–193. 10.1016/j.ijid.2021.02.007. 10.1016/j.ijid.2021.02.007 33578012

[10] Atkins L, Francis J, Islam R (2017). A guide to using the Theoretical Domains Framework of behaviour change to investigate implementation problems. Implement Sci.

[11] Cane J, Richardson M, Johnston M, Ladha R, Michie S (2015). From lists of behaviour change techniques (BCTs) to structured hierarchies: comparison of two methods of developing a hierarchy of BCTs. Br J Health Psychol.

[12] McMillan SS, King M, Tully MP (2016). How to use the nominal group and Delphi techniques. Int J Clin Pharm.

[13] Michie S, Atkins L, West R (2014). The APEASE criteria for designing and evaluating interventions. The behaviour change wheel: a guide to designing interventions.

[14] Razai MS, Osama T, McKechnie DGJ, Majeed A (2021). COVID-19 vaccine hesitancy among ethnic minority groups. BMJ.

[15] Boey L, Bral C, Roelants M (2018). Attitudes, believes, determinants and organisational barriers behind the low seasonal influenza vaccination uptake in healthcare workers - a cross-sectional survey. Vaccine.

[16] Patel A, Sims E, Blacklock J (2022). Cluster randomised control trial protocol for estimating the effectiveness and cost-effectiveness of a complex intervention to increase care home staff influenza vaccination rates compared to usual practice (FLUCARE). Trials.

[17] Moore GF, Audrey S, Barker M (2015). Process evaluation of complex interventions: Medical Research Council guidance. BMJ.

[18] Skivington K, Matthews L, Simpson SA (2021). A new framework for developing and evaluating complex interventions: update of Medical Research Council guidance. BMJ.

[19] Chan A-W, Tetzlaff JM, Gøtzsche PC, Altman DG, Mann H, Berlin J, Dickersin K, Hróbjartsson A, Schulz KF, Parulekar WR, Krleža-Jerić K, Laupacis A, Moher D. SPIRIT 2013 Explanation and Elaboration: Guidance for protocols of clinical trials. BMJ. 2013;346:e7586. 10.1136/bmj.e7586 PMC3541470 23303884

[20] O’Cathain A, Murphy E, Nicholl J (2010). Three techniques for integrating data in mixed methods studies. BMJ.

[21] Holtrop JS, Estabrooks PA, Gaglio B (2021). Understanding and applying the RE-AIM framework: clarifications and resources. J Clin Transl Sci.

