# Supplementary material for: Protocol of the process evaluation of cluster randomised control trial for estimating the effectiveness and cost-effectiveness of a complex intervention to increase care home staff influenza vaccination rates compared to usual practice (FluCare)
Source: Trials. 2023 Sep 15;24:587. doi: 10.1186/s13063-023-07613-5 (PMC10503150; doi:10.1186/s13063-023-07613-5)
Supplement: Supplementary file 1 — Additional file 1. [file 13063_2023_7613_MOESM1_ESM.docx]

# **SPIRIT Checklist for *Trials***

Complete this checklist by entering the page and line numbers where each of the items listed below can be found in your manuscript.

Your manuscript may not currently address all the items on the checklist. Please modify your text to include the missing information. If you are certain that an item does not apply, please state "n/a" and provide a short explanation. **Leaving an item blank or stating “n/a” without an explanation will lead to your manuscript being returned before review.**

Upload your completed checklist as an additional file when you submit to *Trials*. You must reference this additional file in the main text of your protocol submission. The completed SPIRIT figure must be included within the main body of the protocol text and can be downloaded here: http://www.spirit-statement.org/schedule-of-enrolment-interventions-and-assessments/

In your methods section, please state that you used the SPIRIT reporting guidelines, and cite them as:

Chan A-W, Tetzlaff JM, Gøtzsche PC, Altman DG, Mann H, Berlin J, Dickersin K, Hróbjartsson A, Schulz KF, Parulekar WR, Krleža-Jerić K, Laupacis A, Moher D. SPIRIT 2013 Explanation and Elaboration: Guidance for protocols of clinical trials. BMJ. 2013;346:e7586

|  |  | **Reporting Item** | **Page and Line Number** | **Reason if not applicable** |
| --- | --- | --- | --- | --- |
| **Administrative information** | | | | |
| Title | #1 | Descriptive title identifying the study design, population, interventions, and, if applicable, trial acronym | Page 1, lines 1-3 |  |
| Trial registration | #2a | Trial identifier and registry name. If not yet registered, name of intended registry | Page 3, line 66 |  |
| Trial registration: data set | #2b | All items from the World Health Organization Trial Registration Data Set | Page 16 line370-377 |  |
| Protocol version | #3 | Date and version identifier | Page 5 line 128-129 |  |
| Funding | #4 | Sources and types of financial, material, and other support | Page 16, lines 382386 |  |
| Roles and responsibilities: contributorship | #5a | Names, affiliations, and roles of protocol contributors | Page 1-1 lines 4-28  Author contribution page 15 line 349-355 |  |
| Roles and responsibilities: sponsor contact information | #5b | Name and contact information for the trial sponsor | Page 17, line 391 |  |
| Roles and responsibilities: sponsor and funder | #5c | Role of study sponsor and funders, if any, in study design; collection, management, analysis, and interpretation of data; writing of the report; and the decision to submit the report for publication, including whether they will have ultimate authority over any of these activities | Pages 16, lines 368-386 |  |
| Roles and responsibilities: committees | #5d | Composition, roles, and responsibilities of the coordinating centre, steering committee, endpoint adjudication committee, data management team, and other individuals or groups overseeing the trial, if applicable (see Item 21a for data monitoring committee) | In main RCT trial paper referenced in this paper Patel et al. Trials (2022) 23:989  https://doi.org/10.1186/s13063-022-06925-2 |  |
| **Introduction** |  |  |  |  |
| Background and rationale | #6a | Description of research question and justification for undertaking the trial, including summary of relevant studies (published and unpublished) examining benefits and harms for each intervention | Pages 3-5, lines 69-129 |  |
| Background and rationale: choice of comparators | #6b | Explanation for choice of comparators | Page 6-7, line 168-172 | Not true comparators as this is a process evaluation but cover sample size |
| Objectives | #7 | Specific objectives or hypotheses | Page 6, line 147-154 |  |
| Trial design | #8 | Description of trial design including type of trial (eg, parallel group, crossover, factorial, single group), allocation ratio, and framework (eg, superiority, equivalence, non-inferiority, exploratory) | Page 5-6, line 134-166 |  |
| **Methods: Participants, interventions, and outcomes** | | | | |
| Study setting | #9 | Description of study settings (eg, community clinic, academic hospital) and list of countries where data will be collected. Reference to where list of study sites can be obtained | Page 6-7, lines 168-172 |  |
| Eligibility criteria | #10 | Inclusion and exclusion criteria for participants. If applicable, eligibility criteria for study centres and individuals who will perform the interventions (eg, surgeons, psychotherapists) | Pages 6-7 line 168-172 |  |
| Interventions: description | #11a | Interventions for each group with sufficient detail to allow replication, including how and when they will be administered | Pages 6 line 156-166) | Note full details in RCT paper brefily summarized in this Precoess evaluation papers Patel et al. Trials (2022) 23:989  https://doi.org/10.1186/s13063-022-06925-2 |
| Interventions: modifications | #11b | Criteria for discontinuing or modifying allocated interventions for a given trial participant (eg, drug dose change in response to harms, participant request, or improving / worsening disease) | N/A | Not applicable in process evaluation |
| Interventions: adherance | #11c | Strategies to improve adherence to intervention protocols, and any procedures for monitoring adherence (eg, drug tablet return; laboratory tests) | N/A | One of the objectives of the trial is precisely to test the delivery of the intervention in care homes. It would thus be inappropriate to for the research team to develop adherence strategies. The process evaluation will report on fidelity |
| Interventions: concomitant care | #11d | Relevant concomitant care and interventions that are permitted or prohibited during the trial | N/A | These details reported in RCT paper Patel et al. Trials (2022) 23:989  https://doi.org/10.1186/s13063-022-06925-2 |
| Outcomes | #12 | Primary, secondary, and other outcomes, including the specific measurement variable (eg, systolic blood pressure), analysis metric (eg, change from baseline, final value, time to event), method of aggregation (eg, median, proportion), and time point for each outcome. Explanation of the clinical relevance of chosen efficacy and harm outcomes is strongly recommended | N/A | As above in RCT paper Process evaluation will provide explanation for outcomes |
| Participant timeline | #13 | Time schedule of enrolment, interventions (including any run-ins and washouts), assessments, and visits for participants. A schematic diagram is highly recommended (see Figure) | Page 7 line 170 |  |
| Sample size | #14 | Estimated number of participants needed to achieve study objectives and how it was determined, including clinical and statistical assumptions supporting any sample size calculations | Page 5 line 168-172 |  |
| Recruitment | #15 | Strategies for achieving adequate participant enrolment to reach target sample size | N/A | In process evaluation recruited directly from trial population |
| **Methods: Assignment of interventions (for controlled trials)** | | | | |
| Allocation: sequence generation | #16a | Method of generating the allocation sequence (eg, computer-generated random numbers), and list of any factors for stratification. To reduce predictability of a random sequence, details of any planned restriction (eg, blocking) should be provided in a separate document that is unavailable to those who enrol participants or assign interventions | N/A | randomized occurs in trial not process evaluation where purposive sampling is used |
| Allocation concealment mechanism | #16b | Mechanism of implementing the allocation sequence (eg, central telephone; sequentially numbered, opaque, sealed envelopes), describing any steps to conceal the sequence until interventions are assigned | N/A | As above |
| Allocation: implementation | #16c | Who will generate the allocation sequence, who will enrol participants, and who will assign participants to interventions | N/A | As above |
| Blinding (masking) | #17a | Who will be blinded after assignment to interventions (eg, trial participants, care providers, outcome assessors, data analysts), and how | N/A | Researchers in process evaluation not blinded as using purposive sampling |
| Blinding (masking): emergency unblinding | #17b | If blinded, circumstances under which unblinding is permissible, and procedure for revealing a participant’s allocated intervention during the trial | N/A |  |
| **Methods: Data collection, management, and analysis** | | | | |
| Data collection plan | #18a | Plans for assessment and collection of outcome, baseline, and other trial data, including any related processes to promote data quality (eg, duplicate measurements, training of assessors) and a description of study instruments (eg, questionnaires, laboratory tests) along with their reliability and validity, if known. Reference to where data collection forms can be found, if not in the protocol | Page 7 line 173 242 |  |
| Data collection plan: retention | #18b | Plans to promote participant retention and complete follow-up, including list of any outcome data to be collected for participants who discontinue or deviate from intervention protocols | N/A | Recruited from the trial cohort and single interview is data collection method |
| Data management | #19 | Plans for data entry, coding, security, and storage, including any related processes to promote data quality (eg, double data entry; range checks for data values). Reference to where details of data management procedures can be found, if not in the protocol | Page 11, lines 231-235 |  |
| Statistics: outcomes | #20a | Statistical methods for analysing primary and secondary outcomes. Reference to where other details of the statistical analysis plan can be found, if not in the protocol | N/A | In process evaluation data predominantly qualitative any statistical analysis will be descriptive accounts of Likert questionnaire respo0nses see page 10 |
| Statistics: additional analyses | #20b | Methods for any additional analyses (eg, subgroup and adjusted analyses) | Page10 line 210-238  Describes qualitative analysis |  |
| Statistics: analysis population and missing data | #20c | Definition of analysis population relating to protocol non-adherence (eg, as randomised analysis), and any statistical methods to handle missing data (eg, multiple imputation) | N/A |  |
| **Methods: Monitoring** | | | | |
| Data monitoring: formal committee | #21a | Composition of data monitoring committee (DMC); summary of its role and reporting structure; statement of whether it is independent from the sponsor and competing interests; and reference to where further details about its charter can be found, if not in the protocol. Alternatively, an explanation of why a DMC is not needed | As per main trail in RCT paper | Patel et al. Trials (2022) 23:989  https://doi.org/10.1186/s13063-022-06925-2 |
| Data monitoring: interim analysis | #21b | Description of any interim analyses and stopping guidelines, including who will have access to these interim results and make the final decision to terminate the trial | N/A |  |
| Harms | #22 | Plans for collecting, assessing, reporting, and managing solicited and spontaneously reported adverse events and other unintended effects of trial interventions or trial conduct | N/A |  |
| Auditing | #23 | Frequency and procedures for auditing trial conduct, if any, and whether the process will be independent from investigators and the sponsor | N/A | Since the trial is low-risk and very brief (given the length of flu season), there is insufficient time to audit during the trial.  Such audit is not required in a process evaluation |
| **Ethics and dissemination** | | | | |
| Research ethics approval | #24 | Plans for seeking research ethics committee / institutional review board (REC / IRB) approval | Page16, lines 362-366 |  |
| Protocol amendments | #25 | Plans for communicating important protocol modifications (eg, changes to eligibility criteria, outcomes, analyses) to relevant parties (eg, investigators, REC / IRBs, trial participants, trial registries, journals, regulators) | N/A | Reported in main trail  Patel et al. Trials (2022) 23:989  https://doi.org/10.1186/s13063-022-06925-2 |
| Consent or assent | #26a | Who will obtain informed consent or assent from potential trial participants or authorised surrogates, and how (see Item 32) | Page 10 line 221 | For these one off staff interviews electronic consent will be obtained prior to interview and verbal consent confirmed at the beginning of the interviewer by one of three researchers undertaking the interviews |
| Consent or assent: ancillary studies | #26b | Additional consent provisions for collection and use of participant data and biological specimens in ancillary studies, if applicable | N/A |  |
| Confidentiality | #27 | How personal information about potential and enrolled participants will be collected, shared, and maintained in order to protect confidentiality before, during, and after the trial | Page 11 line 232-233 |  |
| Declaration of interests | #28 | Financial and other competing interests for principal investigators for the overall trial and each study site | Page 16 line 379- |  |
| Data access | #29 | Statement of who will have access to the final trial dataset, and disclosure of contractual agreements that limit such access for investigators | Page 16 line370-377 |  |
| Ancillary and post trial care | #30 | Provisions, if any, for ancillary and post-trial care, and for compensation to those who suffer harm from trial participation | N/A | Low risk research, no harm to participants. |
| Dissemination policy: trial results | #31a | Plans for investigators and sponsor to communicate trial results to participants, healthcare professionals, the public, and other relevant groups (eg, via publication, reporting in results databases, or other data sharing arrangements), including any publication restrictions | [N/A | Full dissemination plan in main trail paper. In the process evaluation results will inform next work package page 12 line 273-274 |
| Dissemination policy: authorship | #31b | Authorship eligibility guidelines and any intended use of professional writers | N/A | Reported in main trial paper |
| Dissemination policy: reproducible research | #31c | Plans, if any, for granting public access to the full protocol, participant-level dataset, and statistical code | Page 16 line369-377 |  |
| **Appendices** | | | | |
| Informed consent materials | #32 | Model consent form and other related documentation given to participants and authorised surrogates | N/A |  |
| Biological specimens | #33 | Plans for collection, laboratory evaluation, and storage of biological specimens for genetic or molecular analysis in the current trial and for future use in ancillary studies, if applicable | N/A | No biological specimens taken |

It is strongly recommended that this checklist be read in conjunction with the SPIRIT 2013 Explanation & Elaboration for important clarification on the items. Amendments to the protocol should be tracked and dated. The SPIRIT checklist is copyrighted by the SPIRIT Group under the Creative Commons “Attribution-NonCommercial-NoDerivs 3.0 Unported” license. This checklist can be completed online using https://www.goodreports.org/, a tool made by the EQUATOR Network in collaboration with Penelope.ai
